# Quantification of rutin in rat's brain by UHPLC/ESI-Q-TOF-MS/MS after intranasal administration of rutin loaded chitosan nanoparticles

**DOI:** 10.17179/excli2016-361

**Published:** 2016-08-17

**Authors:** Niyaz Ahmad, Rizwan Ahmad, Atta Abbas Naqvi, Md Aftab Alam, Mohd Samim, Zeenat Iqbal, Farhan Jalees Ahmad

**Affiliations:** 1 Department of Pharmaceutics, College of Clinical Pharmacy, University of Dammam, Dammam-31441, Kingdom of Saudi Arabia; 2 Department of Natural Products and Alternative Medicine, College of Clinical Pharmacy, University of Dammam, Dammam-31441, Kingdom of Saudi Arabia; 3 Department of Pharmacy Practice, College of Clinical Pharmacy, University of Dammam, Dammam 31441, Kingdom of Saudi Arabia; 4 Department of Pharmaceutics, School of Medical and Allied Sciences, Galgotias University, Gautam Budh Nagar, Greater Noida-201310, India; 5 Department of Chemistry, Faculty of Science, Hamdard University, New Delhi-110062, India; 6 Nanomedicine Lab, Department of Pharmaceutics, Faculty of Pharmacy, Jamia Hamdard, Hamdard Nagar, New Delhi-110062, India

**Keywords:** rutin, UHPLC-MS/MS-ESI-Q-TOF, method development and validation, chitosan nanoparticles, brain pharmacokinetic

## Abstract

Rutin (RT), an antioxidant drug, has been utilized to treat cerebral ischemia hence a sensitive quantification method for estimation of RT in brain homogenate is necessary to develop. This study aims to prepare RT loaded Chitosan Nanoparticles (RT-CS-NPs) develop and validate ultra-high performance liquid chromatography-electrospray ionization-synapt mass spectrometric method Synapt Mass Spectrometry (Synapt MS) (UHPLC/ESI-QTOF-MS/MS) for quantification of RT in brain homogenate from Wistar rat. The process of chromatographic separation was carried out on Waters ACQUITY UPLC™ with the components of separation in detail as; column: BEH C-18 with dimension as 2.1 mm×100 mm and particle size 1.7 µm, mobile phase: acetonitrile (85 % v/v/v): 2 mM ammonium formate (15 % v/v/v): formic acid (0.1 % v/v/v) and flow rate: 0.25 mL/min. Liquid-liquid extraction method (LLE), in mixture, i.e. ethyl acetate:acetonitrile, was considered to optimize the recovery of analyte from the brain homogenate of Wistar rat. Over a total run time of 5 minutes, the elution time for RT and internal standard (IS), i.e. Tolbutamide, observed was 2.67 and 2.82 min respectively whereas the transition observed for RT and IS was at m/z 611.1023/303.1071 and 271.1263/155.1073, respectively. Results, regarding various processes and parameters studied for RT as summarized, established a linear dynamic range over a concentration range of 1.00 ng/mL - 1000.0 ng/mL with r^2^; 0.9991±0.0010. Accuracy for intra and inter-assay in terms of % CV revealed a range of 0.45- 2.11 whereas lower limit of detection (LOD) and quantitation (LOQ) observed was 0.09 ng/mL and 0.142 ng/mL, respectively. The analyte stability as well as method specificity and accuracy, i.e. recovery > 86 %, supports the idea for application of current developed method in order to quantify and evaluate the RT-loaded-CS-NPs for RT determination in brain homogenate after intranasal drug delivery.

## Abbreviations

UHPLC-MS/MS: Ultra high performance liquid chromatography mass spectroscopy and mass spectroscopy; Rutin: RT; PPT: Protein precipitation; LLE: liquid-liquid extraction; SPE: solid-phase extraction; LLOQ: Lower limit of quantification; LLOQ QC: Lower limit of quantification for quality control; LQC: Low quality control; MQC: Middle quality control; HQC: High quality control; Q-TOF: Quadrupole Time of Flight; ESI: Electrospray ionization; C_max_: Maximum plasma concentration; K_el_: Elimination rate constant; Tmax: Time to Cmax; t_½_: Half-life; AUC: Area under curve; LOD: lower limit of detection; LOQ: lower limit of quantitation; CS: Chitosan; NPs: Nanoparticles

## Introduction

Rutin, isolated from plants, i.e. *Ruta graveolens *and *Carpobrotus edulis *exhibits potent antioxidant activity. Recently, the research studies on rutin revealed the effectiveness of this drug in treating cerebral ischemia (Annapurna et al., 2013[6]; Zhang et al., 2013[25]; Jang et al., 2014[13]; Rodrigues et al., 2013[18]; Khan et al., 2009[14]; Raza et al., 2011[17]), however a major problem of low water solubility hence low bioavailability still exist with rutin (Park et al., 2013[16]; Veselova et al., 2012[21]; Sasikala et al., 2013[19]). In addition, literature studies report rutin, a drug with lipophilic property (Baldisserotto et al., 2015[7]; Viskupicová et al., 2015[22]) and thus it is a well-known fact that oral delivery of lipophilic drugs including rutin encounters different problems such as i) permeability complications leading to poor bioavailability ii) more prone to chemical and enzymatic degradation in the gastrointestinal tract, and iii) extensive hepatic first-pass metabolism. The complications aforementioned necessitate effective solution in the form of intranasal drug administration for targeted therapy in brain with numerous advantages, i.e. ease of transportation for drug in significant amount into cerebrospinal fluid (CSF) and olfactory bulb, a safe, novel, painless, non-invasive and effective route of drug administration requiring less technical skills and effective for localized therapeutic effects (Ahmad et al., 2013[3], 2015[1]; Wang et al., 2008[23]). Till date different strategies in term of route of administration and formulations have been applied in order to enhance RT bioavailability and we hereby added up a strategy via formulating chitosan nanoparticles loaded with RT and administered intranasal. 

Likewise, literature survey (i.e. Zhang et al., 2010[26]; Chen et al., 2015[10]; Soares et al., 2015[20]; Cen et al., 2015[9]) reports different methods for RT plasma analysis, however, most of these reported studies showed simultaneous development of RT plasma analysis in many drugs at the same time, i.e. no reports of plasma analysis are available for RT individually. Besides, other important reason for current study are lack of research available for brain tissue analysis of RT, unavailability of a sensitive bioanalytical method for estimation of plasma RT analysis alone as well as in brain tissue and above all lack of research studies for estimation of RT at picogram level in any of the plasma or brain tissue. 

Upto the best of our knowledge, current study is a first time study of its kind in order to develop and validate a bioanalytical method for RT encapsulated in CS nanoparticles via UHPLC/ESI-Q-TOF-MS/MS. The developed method showed wide application and more efficiency in terms of high sensitivity and low retention time, for successful bioanalytical investigation in brain as well as brain pharmacokinetics. In addition, current method offers an extra advantage of rutin quantification in brain tissue.

## Materials and Methods

Chitosan, i.e. medium molecular weight with degree of acetylation of 85 %, Glacial acetic acid from IOL Chemical Ltd (Mumbai, India), Sodium tripolyphosphate (TPP), Acetonitrile (LC-MS grade), Formic acid (LC-MS grade) and Methanol (LC-MS grade) were purchased from Sigma-Aldrich (St Louis, MO). Sodium hydroxide (NaOH), 1-octanol, potassium dihydrogen phosphate, Ammonium formate (MS grade) and ammonium acetate (MS grade) as well as Formic acid (purity > 98 %) were purchased from Fluka analytical (Sigma-Aldrich, the Netherland) and Fluka analytical (Germany) respectively. The Milli-Q water purification system, i.e. (Millipore, Bedfrod, MA, USA) was used for purification of Deionized water. A Sigma-Aldrich cellophane tube with dimensions, i.e. Mol. wt. cut-off (12,000 Da), flat (25 mm), diameter (16 mm) and capacity (60 mL/ft) was used in the study.

### Nanoformulation development of Chitosan nanoparticles (Ionic Gelation Method)

Chitosan nanoparticles (CS-NPs) were prepared via ionic gelation techniques as reported (Calvo et al., 1997[8]; Aktas et al., 2005[5]). Placebo NPs were prepared initially which was attained as dropwise addition of aqueous solution of TTP (0.15 %) with a solution of CS (0.15 %) while continuously stirring at room temperature. The mechanism behind formulation of placebo CS-NPs was ionic interaction between positively charged amino group form CS and negative groups of TTP, the final ratio for which was established on the basis of preliminary studies. The same procedure was utilized for the preparation of RT-loaded CS-NPs, keeping the ratio for CS/TTP unchanged while the ratio for RT was varied in order to observe the effect of initial RT concentration upon the characteristics as well as the *in-vitro* release profile of NPs. Followed by centrifugation for 30 minutes at 4000 rpm and 4 °C and collection of CS-NPs after supernatant is discarded.

### Characterization of nanoparticles

#### Particle size and zeta-potential measurements

For the determination of particle size a Nano-series Zetasizer (Nano-ZS, HAS 3000, Malvern Instruments Ltd, Worcestershire, UK), based on the principle of photon correlation spectroscopy, was used. In detail for the determination of zeta potential all the samples (NPs) were diluted properly with Milli-Q water (the dispersant dielectric constant value for water set as 78.5) and the electrophoretic mobility was obtained at 25 °C which was calculated finally with the help of DTS (version 4.1) software from (Malvern, Worcestershire, UK). 

#### Transmission electron microscopy (TEM) 

The surface morphology for prepared CS-NPs was determined with the help of TEM (Morgagni 268D; FEI Company, Hillsboro, OR). In detail one drop of nanosuspension was put on a paraffin sheet successively followed by covering with a copper grid, keeping for a time period of one (01) minute in order for the NPs to stick and at the end keeping the grid for a time of > 5 seconds on one drop of phosphotungstate. The samples, after clearing the remaining solution with the help of filter paper, were air dried and observed again with TEM.

#### Scanning electron microscopy (SEM) 

The surface texture for the optimized RT-CS-NPs was confirmed with the help of SEM (Zeiss EVO40; Carl Zeiss, Cambridge, UK). In detail the sample was spread on a double-sided conductive tape successively followed with stucking, under high vacuum with gold, in SCD020 Blazers sputter coater unit (BAL-TEC GmbH, Witten, Germany) where the environment was already maintained with argon gas (50 mA) for 100 seconds.

#### UHPLC conditions

Waters ACQUITY UPLC^TM^ system from Waters Corp., MA, USA, hyphenated with a binary system of solvent delivery and tuneable MS detector (Synapt; Waters, Manchester, UK), was used to perform the process of UHPLC whereas Waters ACQUITY UPLC^TM^ BEH C-18 column with dimension, i.e. 2.1 mm × 100 mm, 1.7 µm was utilized for chromatographic separation. The UHPLC mobile phase conditions were as follows: degassed LC-MS grade solvent, i.e. Acetonitrile (85 %): 10 mM Ammonium Formate (15 %): Formic Acid (0.1 %), v/v/v, with isocratic elution, flow rate of 0.25 mL/min sample injection volume of 10 µl/run and a total chromatographic run time of 5.0 min.

#### ESI-Q-TOF-MS conditions 

**Figure 1:**
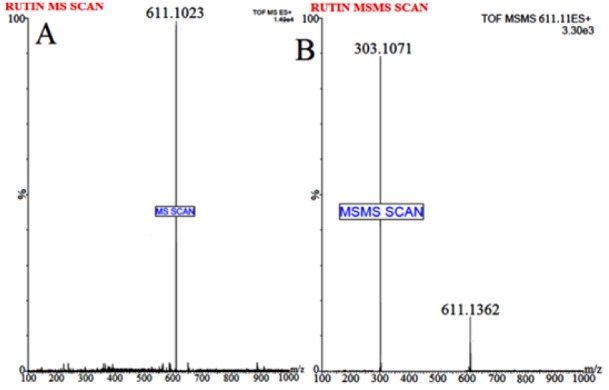
Mass spectrum of (A) Rutin parent/protonated molecule at m/z 611.1023) and (B) Rutin product ion (major fragmented product ion at m/z 303.1071) showing fragmentation transitions

**Figure 2:**
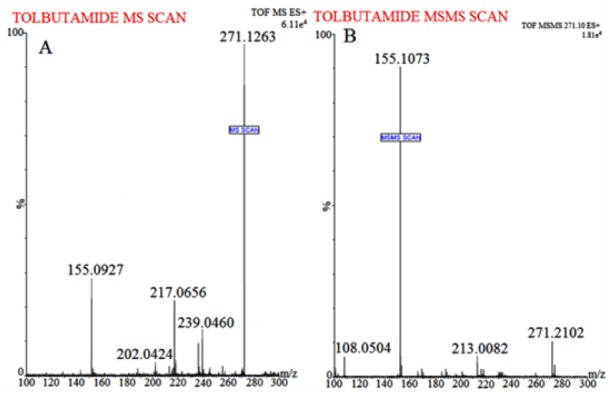
Mass spectrum of (A) Tolbutamide (IS) protonated molecule at m/z 271.1263 and (B) IS product ion (major fragmented product ions at m/z 155.1073) showing fragmentation transitions

Waters Q-TOF Premier mass spectrometer system (Micromass MS Technologies, Manchester, UK) was used to perform MS. The Q-TOF Premier TM operating conditions were as follows: V mode operation with resolution over 32000 mass and scan time of 1.0 min whereas inter-scan delay of 0.02 sec along with a collision gas, i.e. argon at a pressure 5.3×10^-5^ Torr. Synapt Mass Spectrometry (Synapt MS) used for quantification (set for trap collision energy (Trap CE) at 26.8 and 24.64 eV) showed transitions, i.e. RT at m/z 611.1023 and Tolbutamide (IS) at m/z 271.1263, as shown in Figures 1(Fig. 1) and 2(Fig. 2). Mass Lynx software V 4.1 was used to calculate the accurate mass and composition for the precursor and fragment ions.

#### Quality Control (QC) sample and standard sample preparation

The requisite amount of RT was dissolved in methanol and sonicated for 20 minutes at 44 kHz/250W in order to prepare a standard stock solution of 10 mg/mL for RT. A set of eight non-zero concentrations (A-H) for calibration curve (CC) standards were prepared as spiking aqueous analyte (2 %) in blank rat brain homogenate, i.e. 20 ml (aqueous aliquots) + 980 ml (blank rat brain homogenate) which yielded a concentration range for RT, i.e. 1-1000 ng/mL. For each analyte the final concentration ended up with 1, 2, 25, 210, 420, 640, 850 and 1000 ng/mL. QC samples were prepared independently at four levels as HQC (high quality control) of 800 ng/mL, MQC (middle quality control) of 400 ng/mL, LQC (low quality control) of 2.9 ng/mL and LLOQC of 1.01 ng/mL concentration. A working solution of internal standard (50 ng/mL) was prepared by diluting the stock solution in mixture of methanol and water (1:1). Until use all the solutions were stored at 2-8 °C.

#### Sample preparation protocol

For sample preparation all the solutions, i.e. CC standards, QC samples and unknown brain homogenate samples were prepared freshly. In detail, in a glass tube 600 µl aliquot of each sample + 50 µl (50 ng/mL) of IS was taken and added up with 5 % formic acid (200 µL) solution (for breaking of protein binding) successively followed by vortex for 5 minutes at 300 rpm. 5 ml of extraction mixture, i.e. ethyl acetate (650 mL) and acetonitrile (350 mL) prepared separately, was added and maintained at reciprocating shaker (20 minutes at 100 rpm). The tubes were made to spin in centrifuge (ten minutes at 4000 rpm and 4 ºC) and 4 mL of supernatant organic layer was transferred to another clean glass tube followed by drying in nitrogen stream at pressure NMT 20 psi and temperature 50 ± 2.0 °C. Finally, dried elute was reconstituted in mobile phase (600 µl) and samples prepared were transferred to small vials for injection (10 µL) and analysis. 

#### Bioanalytical method validation

FDA (2001[12]) as reported (Ahmad et al., 2014[4], 2015[1]; Mustafa et al., 2013[15], Faiyazuddin et al., 2012[11]; Wilson et al., 2005[24]) were followed for the bioanalytical method validation of RT in brain homogenate whereas method linearity was determined by analysis of three standard plots which contains eight non-zero concentrations. For the construction of calibration curve, ratio of peak area from analyte/IS were considered, via weighted (1/x^2^) linear least squares regression of the brain concentrations and the measured peak area ratios. The lower limit of quantification (LLOQ) is the lowest concentration of the calibration curve, which could be measured with acceptable accuracy and precision. The lower limit of quantification (LLOQ) was determined on the basis of signal-to-noise ratio (10:1) whereas RT extraction efficiency (recovery), evaluated via comparison between mean area response of six replicates of extracted samples (spiked before extraction) to that of extracted drug free brain homogenate samples (spiked after extraction), was performed at individual level of LQC, MQC and HQC levels. A like, the recovery of IS too was estimated. The replicate analysis of RT brain samples was performed on the same day in order to determine intra-day accuracy and precision. Six replicates of LLOQC, LQC, MQC, and HQC samples and calibration curve were included in the run. For the assessment of inter-day accuracy and precision, separated six precision and accuracy batches on three consecutive days were analysed whereas slight changes in operating conditions (mobile phase composition, pH and flow rate) using LQC, MQC and HQC levels of QC samples were adopted for determination of robustness, however, in order to evaluate ruggedness of the method, one batch of precision and accuracy, using different column (within the same manufacturer) run on the same instrument with the help of different analysts, was made to run. A total of six replicates for LLOQC, LQC, MQC, and HQC samples were run.

#### Matrix effect

Six samples, using six different brain homogenate batches, were prepared at LQC and HQC level in order to study the effect of matrix on quantification of analyte which was checked for %accuracy and precision (%CV) in both QC samples. In detail, back calculated values from QC's nominal concentration were used to assess matrix effect. Following the specified storage condition for samples the analysis was performed and matrix effect was investigated via post extraction spike method. The comparison for Peak area (A) of the analyte (known concentration of spiked blank brain homogenate (MQC)) with corresponding peak area (B) (obtained via direct injection of standard in the mobile phase) was performed whereas the ratio, i.e. A/B×100, is defined as the matrix effect.

#### LOD and LOQ

The standard deviation responses (SD) for the triplicate mobile phase blank injections along with slope of calibration (S) was used to calculate LLOD and LLOQ. Successively followed, LOD and LOQ were experimentally determined as dilute known concentrations of RT until the average responses were approximately 3 or 10 times the standard deviation of the responses for triplicate. The following formula was used to determine the values for LOD and LOQ:


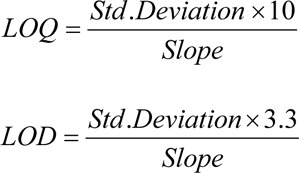


#### Ex vivo stability

Six replicates, at concentrations of (LQC) 2.9 ng/mL and (HQC) 800 ng/mL and exposed to different conditions of time and temperature, from brain homogenate were analysed to evaluate the stability of RT. Percentage stability was determined as:


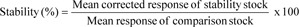


#### Long-term stability

Six replicates of LQC and HQC, stored for one month at deep freezer (-80 °C), standard spiked brain homogenate sample were assessed for long term stability.

#### Freeze-thaw stability

Six replicates of LQC and HQC, undergone three consecutive freeze thaw cycles, i.e. from -20 °C to room temperature (+ 25 °C), brain homogenate were considered for evaluation in freeze-thaw stability studies. 

#### Bench-top stability

After storage for 24 h in optimized conditions, six set each of LQC and HQC were used to evaluate bench-top stability whereas QC samples were quantified against freshly spiked calibration curve standards.

#### Post-processing stability

Six set each of LQC and HQC of processed samples, exposed to a temperature of 10 °C in an autosampler for 24 h, were considered to determine the short term stability. Following specified storage conditions, sample were processed and analysed with conclusion, analyte exhibiting a precision (below 15 %) and accuracy (85-115 %) are stable (Ahmad et al., 2014[4]; Mustafa et al., 2013[15]). 

### In vivo study

#### Experimental animal

The experimental animal study was performed after proper approval (protocol approval No. 847), Animal Ethical Committee, Jamia Hamdard (New Delhi, India) which ensures to confirm according to National Guidelines on the Care and Use of Laboratory Animals. One week before experiments; Wistar rats (n:6, weight: 300-400 g and age: 8-10 weeks) were maintained in an environment with controlled room temperature (25 ± 2 °C) and humidity (60 ± 5 %) for 12 h dark-light cycle. In addition standard pelleted diet and water were used to feed animals. Before the experiment the rats were kept on fasting overnight. 

#### Experimental protocol

This study used animal protocol already approved by Institutional Animal Ethics Committee, Jamia Hamdard. Prior to pharmacokinetic studies, rats were fasted for 12 h. The CS-Nanoparticles formulations (0.15 %:0.15 %::CS/TPP (v/v)) separately, encapsulating RT in a concentration of 10 mg/kg after intranasal administration were quantified in Wistar rat striatum via bioanalytical method. Rats from different groups, i.e. control, API solution and nanoparticles treated, were sacrificed after 1 h in order to isolate striatum followed by homogenization with phosphate buffer (10 % w/v; pH 7.4), centrifugation (2500 × g) for 10 minutes at 20 °C and subsequent collection of supernatant fractions. Until analysis collected brain striatum samples were preserved at -80 °C for further investigation. 

## Results and Discussion

The flavonoid RT is considered a low molecular weight compound, i.e. MW 610.52 with an alcoholic group present in the structure which imparts it the sensitivity to be easily detected in positive ion mode. Various solvents, i.e. methanol and isopropyl alcohol were tried for mobile phase selection but they didn't provide efficient chromatographic resolution. In addition, amongst the buffer system studied, ammonium formate (15 % v/v): formic acid (1 % v/v) buffer system resulted in sharp peak and efficient signal response. Following different in-depth trials with change of solvents and buffer conditions etc., optimum chromatographic system for separation of RT achieved was as follows: mobile phase: Acetonitrile (85 % v/v): 2mM Ammonium Formate (15 % v/v): Formic Acid (0.1 % v/v); flow rate: 0.25 mL/min and run time of 5.0 minutes (provided a baseline separation for RT and IS without interference). The full-scan MS spectra for RT revealed, protonated molecule at m/z 611.1023 as shown in Figure 1(Fig. 1) whereas, during direct infusion, IS mass spectra showed protonated molecule at m/z 271.1263 (Figure 2(Fig. 2)). The optimum collision energies employed were 26.8 eV (RT) and 24.64 eV (IS) whereas capillary voltage of 4.5 kV was used in order to monitor precursor ions.

**Figure 3:**
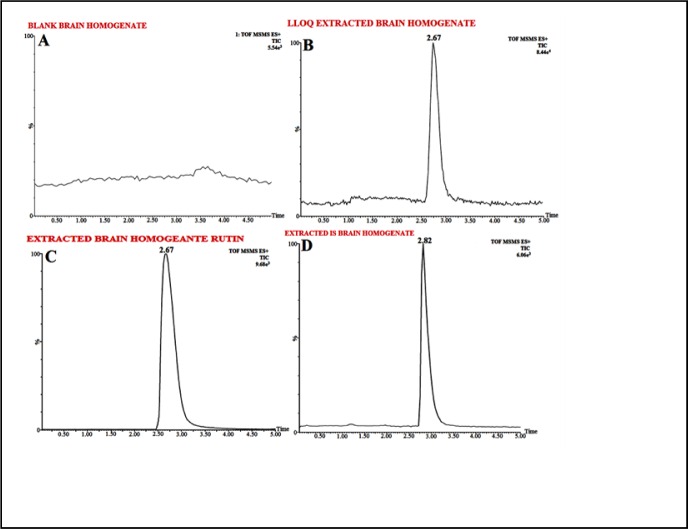
Typical chromatograms of (A) Extracted Blank Brain Homogenate, (B) LLOQ Extracted Brain Homogenate Rutin, (C) Extracted Brain Homogenate Rutin, (D) Extracted Brain Homogenate Tolbutamide IS (100 ng mL^-1^) extracted after spiking with Wistar rat-brain homogenate by selective reaction monitoring scan mode.

The biological sample preparation techniques most widely used are Protein precipitation (PPT), liquid-liquid extraction (LLE) and solid-phase extraction (SPE). In detail, initially PPT method was applied for method development but due to strong ion suppression of the endogenous substance in brain homogenate PPT separation was not considered further for API separation. Although, aforementioned problem may be resolved with chromatographic separation but it will lead to run time sacrifice. Following different procedures, LLE method was finally found to be the efficient for preparing RT striatum samples. In order to achieve obtain optimum recovery, seven organic extraction solvents were evaluated, i.e. ethyl acetate, chloroform, dichloromethane, acetonitrile, diethyl ether and tertiary butyl methyl ether (TBME), and n-hexane. To conclude, no solvent alone yielded the highest recovery alone except the extraction mixture, i.e. Ethyl Acetate (650 mL) and acetonitrile (350 mL) which showed the highest recovery of > 86 %for RT and IS. Figure 3A(Fig. 3) represents chromatogram for brain homogenate, i.e. blank (extracted and reconstituted) while Figure 3B-D(Fig. 3) shows elution time for spiked RT brain homogenate sample and IS (50 ng/mL), i.e. at 2.67 min and 2.82 min respectively.

### Preparation of optimized RT-CS-NPs

**Figure 4:**
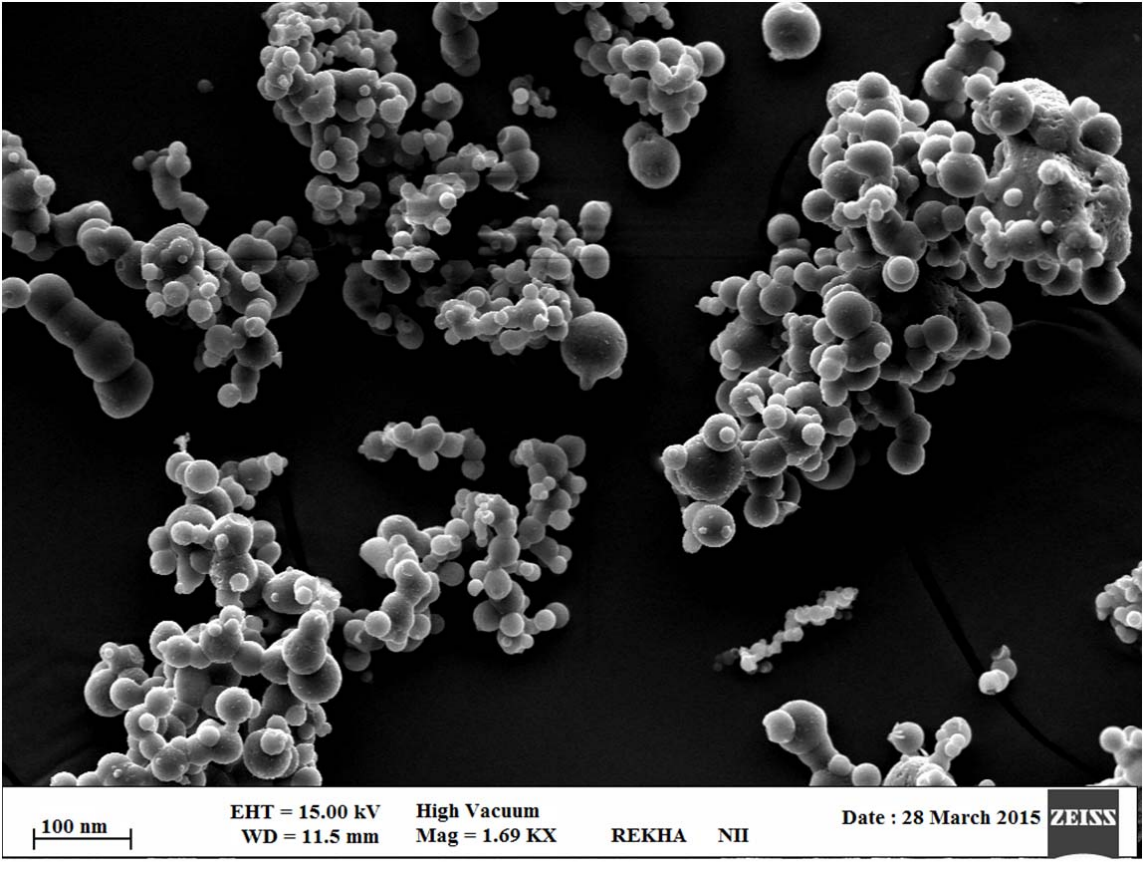
Scanning electron microscopy images (SEM) of surface morphology of the prepared RT-CS-NPs

**Figure 5:**
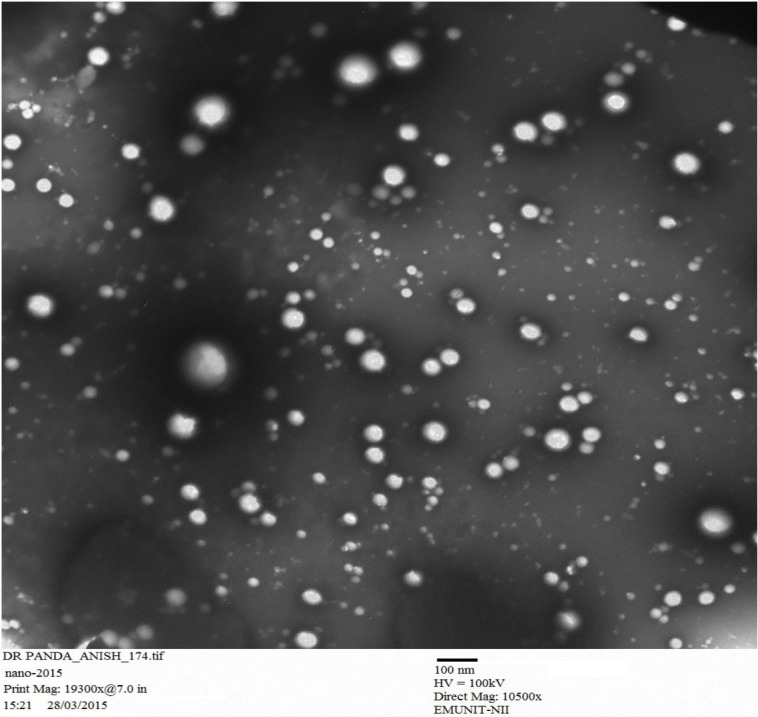
Transmission electron microscopy (TEM) image of CS encapsulated with a rutin of optimized NPs

More than 53 different concentration ratio were evaluated for CS:TTP which showed a final concentration of ratio of (0.15 %:0.15 %) CS:TTP for the optimized nanoparticles (Data not shown in this paper). Different advance and delicate techniques such as TEM and SEM were utilized to evaluate the size and shape of CS-NPs. SEM revealed a round and smooth surface morphology as shown in Figure 4(Fig. 4) whereas TEM showed sphericity and a particle size within range of 85-100 nm as shown in Figure 5(Fig. 5), for RT-CS-NPs. The particle size, is although within the range of optimum particle size for intranasal brain drug delivery as reported (Ahmad et al., 2013[3], 2016[2]), still other parameters were studied in order to optimize our formulation, i.e. TTP concentration, CS concentration, pH and stirring speed in order to obtain small particles, maximum loading with maximum encapsulation efficiency as well as PDI (0.206). Finally, optimized CS-NPs with properties such as optimum particle size, loading capacity, and entrapment efficiency along with a sustained* in vitro* drug release profile of over 24 h were formulated via ionotropic gelation method which was further subjected to Differential Scanning Calorimetry (DSC) for entrapment of drug (Data not shown in this paper).

### Bioanalytical method validation

#### Linearity

The RT calibration curve showed linearity at concentration range of 1-1000 ng/mL with least squares regression r^2^ ≥ 0.99 as well as accuracy (0.45-2.11 %) and precision (95.05-98.62 %.) ( %CV) for RT calibration curve standards. 

#### Accuracy and precision

There was no interference of any endogenous peak from any of the batches with the retention time of analyte or IS. The selectivity of method is demonstrated via representative chromatogram from blank brain extracted homogenate fortified with IS as well as blank brain homogenate fortified with RT as shown in Figure 3A(Fig. 3). The mean recovery for RT, i.e. spiked brain homogenate (n=6) observed at different level was as LQC (85.31 %), MQC (86.38 %) and HQC (87.01 %) whereas the IS recovery was 79.28 %. For all the samples of RT at QC levels, the intra-batch and inter-batch precision (% CV) was in the ranges, i.e. 0.45-1.82 % and 0.52-2.11 %, respectively whereas intra-batch and inter-batch accuracy result were 98.01-98.62 % and 95.05-98.57 %, respectively as shown in detail in Table 1(Tab. 1).

#### Robustness

The suitability of system was evaluating by observing the effects of different parameters on the %age CV and recovery of RT at varying conditions as mobile phase ratio: Acetonitrile: Ammonium Formate (2 mM): Formic Acid at (84.9:14.9:0.09), (85:15:0.1) and (85.9:15.1:0.19) v/v/v concentration, mobile phase pH at 5.9, 6.1 and 6.3 and flow rate: 0.249, 0.250 and 0.260 mL/min. To conclude, robustness of the method was proved by obtaining low values for %CV of RT, i.e. 0.16 - 4.85 %) after small deliberate modifications in the already developed method of UHPLC as shown in Table 2A(Tab. 2).

#### Ruggedness

For ruggedness one complete batch of RT was processed and analysed for precision and accuracy with the help of different analysts while utilizing different column and sets of solutions. The mean %accuracy and % correlation of variance for drugs (n = 6) showed a range of 95.04 - 98.49 and 0.62 - 3.12, respectively as shown in Table 2B(Tab. 2).

#### Matrix effect

The co-elution of some endogenous components present as normal part of biological sample produces matrix effects observed as interference with peak retention from its actual expected position. The matrix effect for RT calculated as (A/B×100) revealed at different level was as LQC (% CV 3.85; n = 6 each) and HQC (% CV 3.28; n = 6 each), 1.36 % and 0.16, respectively. The % value for CV < 5 proves lack of matrix effects upon method. Using formic acid (5 %) as protein precipitating agent, RT showed no significant ion suppression or enhancement in post-column infusion experiments as done with LLE. 

#### LOD and LOQ

LOD and LOQ as estimated after experimental analysis of spiked brain homogenate samples serially diluted with RT standard (until the ratio for signal-to-noise reaches 3 and 10) resulted the value 0.09 ng/mL for LOD and 0.142 ng/mL for LOQ.

#### Ex vivo stability

Table 3(Tab. 3) for *ex vivo* stability results shows the stability of RT over all the storage conditions, i.e. freeze-thaw, long term, post-processing and bench-top stability. Two levels of quality control, i.e. LQC and HQC were considered for investigation of analyte stability in brain homogenate samples and the analyte recovery with respect to time zero was reviewed. In detail the stability results for %RT recovery were as recovery of 98.94 % for LQC and 98.89 % for HQC after long term stability, i.e. 1 month: recovery for LQC and HQC in the range of 98.59 - 97.87 % nd 99.74-99.14 respectively after freeze-thaw stability, i.e. after 1, 2 and 3 freeze thaw cycles: a recovery of 99.29 % for LQC and 99.22 % for HQC observed with bench-top stability, i.e. 24 h and a recovery of 99.65 % for LQC and 98.88 % for HQC as observed after post-processing stability.

#### In vivo study

**Figure 6:**
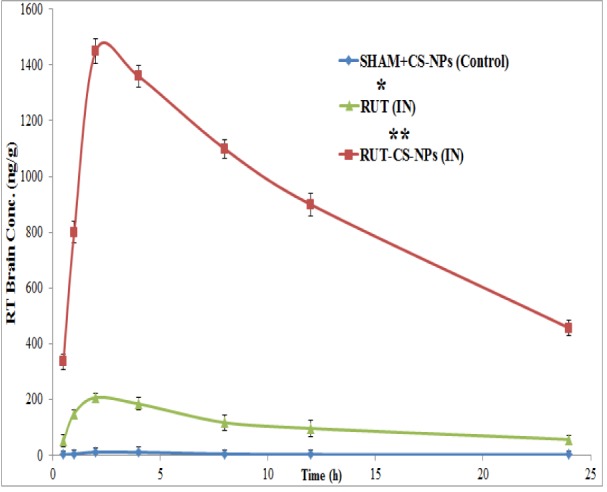
Pharmacokinetic plot of Rutin solution and Rutin loaded CS-NPs in brain after intranasal administration at different time intervals. Significance for Rutin solution and Rutin loaded CS-NPs was determined as *p<0.05 and **p<0.01 when compared with SHAM+CS-NPs group (Placebo control group)

In order to estimate the concentration of RT in Wistar rats striatal tissue, the developed and validated UHPLC/ESI-Q-TOF-MS/MS method as described in this article was applied which resulted as a successful application in order to find mean RT concentration value (ng/mL) in striated waiter rats tissue already treated with RT-CS-NPS via i.n administration (as shown in Figure 6(Fig. 6)). To conclude the quantified results were as SHAM+CS-NPs, i.e. control group showed a value of 10.22 ± 0.0221 ng/mg of protein: the group treated with RT-CS-NPs showed maximum concentration level, i.e. 1449.33 ± 44.88** ng/mg with P < 0.01 whereas maximum concentration for RT solution was 206.212 ± 14.99* ng/mg with P < 0.05. This high significant difference for RT concentration proves the advantage and effectiveness of i.n route of drug administration for drug delivery into brain.

## Conclusion

The rapid, selective and sensitive properties and above all the detection limit upto picogram level supports the potential of the develop method for quantification of RT via UHPLC/ESI-Q-TOF-MS/MS in rat brain homogenate The recovery for three analytes in brain homogenate after extraction procedures, i.e. >86 % as well as the results obtained for accuracy and precision, linearity and stability, i.e. bench-top, long term, freeze thaw stability and post processing stability as well as matrix effect were found within acceptable range of limits. In addition, the developed quantification method was applied successfully for in vivo studies in brain homogenate from Wistar rat's brain with acceptable accuracy and precision range and adequate sensitivity hence supporting the fact, the developed quantification method is efficiently applicable in further clinical studies.

## Declaration of conflict of interests

No conflict of interest exists among authors.

## Authors’ contribution

Niyaz Ahmad and Md Aftab Alam conducted the development and validation of the method, preparation and optimization of RT-CS-NPs manuscript writing; Rizwan Ahmad and Atta Abbas Naqvi performed the pharmacokinetic study and rat samples analysis. Mohd Samim, Zeenat Iqbal, and Farhan Jalees Ahmad designed the study, arranged all chemicals, drugs, polymers and reference standard, and also assisted in method validation and manuscript writing. All authors have read and approved the final version of the manuscript.

## Declaration of grant

No grants were received.

## Acknowledgements

I (Dr. Niyaz Ahmad) am grateful to Prof. (Dr.) Farhan Jalees Ahmad for the collaboration research study in between University of Dammam, Dammam, Saudi Arabia and Jamia Hamdard (Hamdard University), New Delhi, India. 

## Figures and Tables

**Table 1:**
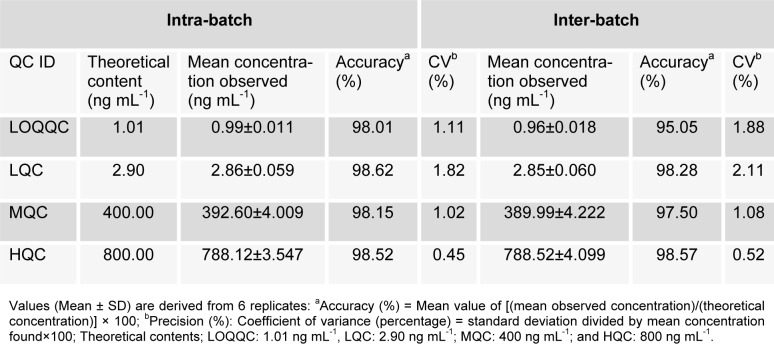
Precision and accuracy data for rutin

**Table 2:**
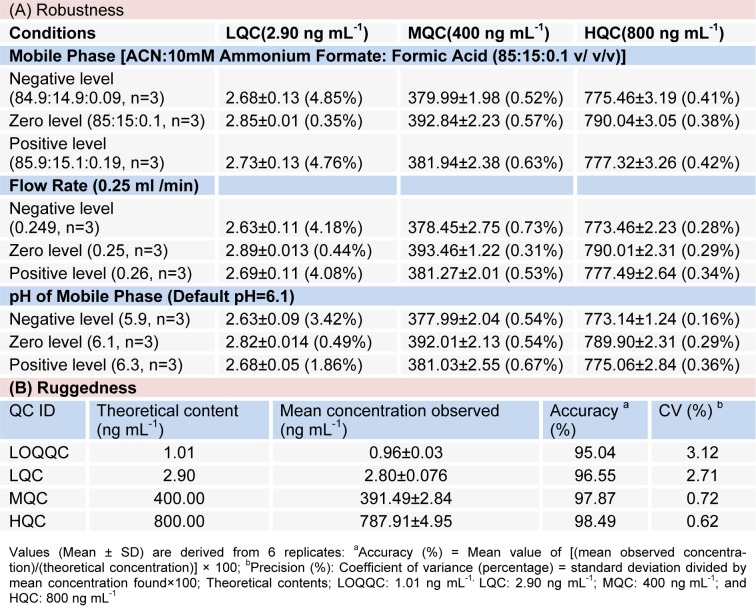
Robustness of the method for rutin

**Table 3:**
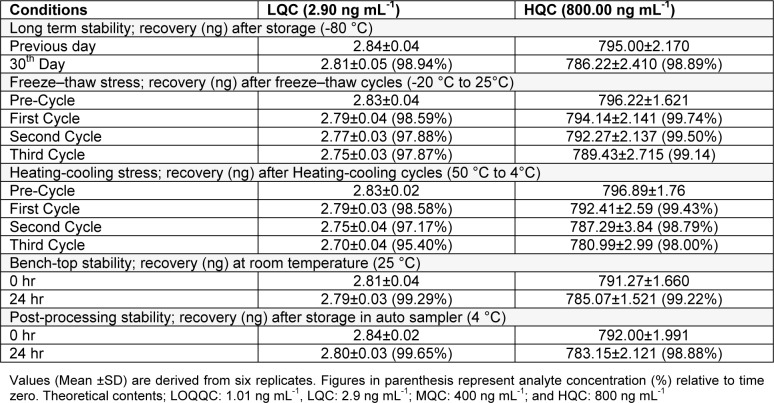
Ex vivo stability data for rutin

## References

[1] Ahmad N, Ahmad I, Umar S, Iqbal Z, Samim M, Ahmad FJ (2015). PNIPAM nanoparticles for targeted and enhanced nose-to-brain delivery of curcuminoids: UPLC/ESI-Q-TOF-MS/MS-based pharmacokinetics and pharmacodynamic evaluation in cerebral ischemia model. Drug Deliv.

[2] Ahmad N, Ahmad R, Alam MA, Samim M, Iqbal Z, Ahmad FJ (2016). Quantification and evaluation of thymoquinone loaded mucoadhesive nanoemulsion for treatment of cerebral ischemia. Int J Biol Macromol.

[3] Ahmad N, Umar S, Ashafaq M, Akhtar M, Iqbal Z, Samim M (2013). A comparative study of PNIPAM nanoparticles of curcumin, demethoxycurcumin, and bisdemethoxycurcumin and their effects on oxidative stress markers in experimental stroke. Protoplasma.

[4] Ahmad N, Warsi MH, Iqbal Z, Samim M, Ahmad FJ (2014). Quantification of curcumin, demethoxycurcumin, and bisdemethoxycurcumin in rodent brain by UHPLC/ ESI-Q-TOF-MS/MS after intra-nasal administration of curcuminoids loaded PNIPAM nanoparticles. Drug Test Anal.

[5] Aktas Y, Andrieux K, Alonso MJ, Calvo P, Gürsoy RN, Couvreur P (2005). Preparation and in vitro evaluation of chitosan nanoparticles containing a caspase inhibitor. Int J Pharm.

[6] Annapurna A, Ansari MA, Manjunath PM (2013). Partial role of multiple pathways in infarct size limiting effect of quercetin and rutin against cerebral ischemia-reperfusion injury in rats. Eur Rev Med Pharmacol Sci.

[7] Baldisserotto A, Vertuani S, Bino A, De Lucia D, Lampronti I, Milani R (2015). Design, synthesis and biological activity of a novel Rutin analogue with improved lipid soluble properties. Bioorg Med Chem.

[8] Calvo P, Remunan-Lopez C, Vila-Jata JL, Alonso MJ (1997). Chitosan and chitosan: ethylene oxide-propylene oxide block copolymer nanoparticles as novel carriers for proteins and vaccines. Pharm Res.

[9] Cen M, Ruan J, Huang L, Zhang Z, Yu N, Zhang Y (2015). Simultaneous determination of thirteen flavo-noids from Xiaobuxin-Tang extract using high-performance liquid chromatography coupled with electrospray ionization mass spectrometry. J Pharm Biomed Anal.

[10] Chen M, Zhang X, Wang H, Lin B, Wang S, Hu G (2015). Determination of rutin in rat plasma by ultra performance liquid chromatography tandem mass spectrometry and application to pharmacokinetic study. J Chromatogr Sci.

[11] Faiyazuddin M, Ahmad N, Iqbal Z, Talegaonkar S, Bhatnagar A, Khar RK (2012). Stabilized terbutaline submicron drug aerosol for deep lungs deposition: Drug assay, pulmonokinetics and biodistribution by UHPLC/ESI-q-TOF-MS method. Int J Pharm.

[12] FDA (2001). Guidance for Industry Bioanalytical Method Validation. FDA.

[13] Jang JW, Lee JK, Hur H, Kim TW, Joo SP, Piao MS (2014). Rutin improves functional outcome via reducing the elevated matrix metalloproteinase-9 level in a photothrombotic focal ischemic model of rats. J Neurol Sci.

[14] Khan MM, Ahmad A, Ishrat T, Khuwaja G, Srivastawa P, Khan MB (2009). Rutin protects the neural damage induced by transient focal ischemia in rats. Brain Res.

[15] Mustafa G, Ahmad N, Baboota S, Ali J, Ahuja A (2013). UHPLC/ESI-Q-TOF-MS method for the measure-ment of dopamine in rodent striatal tissue: a comparative effects of intranasal administration of ropinirole solution over nanoemulsion. Drug Test Anal.

[16] Park SN, Lee MH, Kim SJ, Yu ER (2013). Preparation of quercetin and rutin-loaded ceramide liposomes and drug-releasing effect in liposome-in-hydrogel com-plex system. Biochem Biophys Res Commun.

[17] Raza SS, Khan MM, Ahmad A, Ashafaq M, Khuwaja G, Tabassum R (2011). Hesperidin ameliorates functional and histological outcome and reduces neuroinflammation in experimental stroke. Brain Res.

[18] Rodrigues AM, Marcilio Fdos S, Frazão Muzitano M, Giraldi-Guimarães A (2013). Therapeutic potential of treatment with the flavonoid rutin after cortical focal ischemia in rats. Brain Res.

[19] Sasikala V, Rooban BN, Sahasranamam V, Abraham A (2013). Rutin ameliorates free radical mediated cataract by enhancing the chaperone activity of α-crystallin. Graefes Arch Clin Exp Ophthalmol.

[20] Soares MS, da Silva DF, Forim MR, da Silva MF, Fernandes JB, Vieira PC (2015). Quantification and localization of hesperidin and rutin in Citrus sinensis grafted on C. limonia after Xylella fastidiosa infection by HPLC-UV and MALDI imaging mass spectrometry. Phytochemistry.

[21] Veselova IA, Malinina LI, Rodionov PV, Shekhov-tsova TN (2012). Properties and analytical applications of the self-assembled complex {peroxidase-chitosan}. Talanta.

[22] Viskupicova J, Majekova M, Horakova L (2015). Inhibition of the sarco/endoplasmic reticulum Ca2+-ATPase (SERCA1) by rutin derivatives. J Muscle Res Cell Motil.

[23] Wang X, Chi N, Tang X (2008). Preparation of estradiol chitosan nanoparticles for improving nasal absorption and brain targeting. Eur J Pharm Biopharm.

[24] Wilson ID, Nicholson JK, Castro-Perez J, Granger JH, Johnson KA, Smith BW (2005). High resolution “ultra performance” liquid chromatography coupled to q-TOF mass spectrometry as a tool for differential metabolic pathway profiling in functional genomic studies. J. Proteome Res.

[25] Zhang S, Qi Y, Xu Y, Han X, Peng J, Liu K (2013). Protective effect of flavonoid-rich extract from Rosa laevigata Michx on cerebral ischemia-reperfusion injury through suppression of apoptosis and inflammation. Neurochem Int.

[26] Zhang W, Xu M, Yu C, Zhang G, Tang X (2010). Simulta-neous determination of vitexin-4''-O-glucoside, vitexin-2''-O-rhamnoside, rutin and vitexin from hawthorn leaves flavonoids in rat plasma by UPLC-ESI-MS/MS. J Chromatogr B.

